# Impact of Chronic Organ Disorder Staging on Hospitalization Risk in Elderly Patients With Multimorbidity: A Retrospective Cohort Study

**DOI:** 10.7759/cureus.71168

**Published:** 2024-10-09

**Authors:** Yudai Tanaka, Ryuichi Ohta, Chiaki Sano

**Affiliations:** 1 Community Medicine Management, Shimane University Faculty of Medicine, Izumo, JPN; 2 Community Care, Unnan City Hospital, Unnan, JPN

**Keywords:** aged population, chronic organ disorder, family medicine, general medicine, hospitalization, multimorbidity, retrospective studies, risk factors, rural

## Abstract

Introduction

Multimorbidity, defined as the coexistence of two or more chronic conditions, is increasingly prevalent in the aging population and is linked to adverse health outcomes, including hospitalization. Chronic organ disorder (COD) is a proposed framework in Japan to assess multimorbidity by categorizing organ dysfunction stages. This study investigates the association between COD stages and hospitalization rates in elderly patients with multimorbidity.

Method

A retrospective cohort study was conducted at the Internal Medicine Department of Unnan Municipal Hospital. Patients aged 18 and older with multimorbidity, defined as having two or more chronic diseases, who visited the clinic from April to September 2022 were included. COD stages were classified from A to E, representing increasing severity. Logistic regression analysis was used to evaluate hospitalization-related factors, including age, BMI, serum albumin, and COD stages.

Results

Among 568 patients, 66 (11.6%) were hospitalized. Hospitalized patients were older (75.5 vs. 58.8 years, p < 0.001), had lower BMI (20.65 vs. 22.21 kg/m², p = 0.003), and lower serum albumin levels (3.70 vs. 3.96 g/dL, p < 0.001). Advanced COD (Stage B or above) was associated with an increased risk of hospitalization (OR: 1.86, 95% CI: 1.01-3.45, p = 0.048). Age ≥75 years (OR: 2.93, 95% CI: 1.55-5.54, p < 0.001) and low BMI were also significant predictors.

Conclusion

This study demonstrates that advanced COD stages, age, and low BMI are significant predictors of hospitalization in patients with multimorbidity. Comprehensive COD assessment aids in identifying high-risk patients and potentially guides preventive interventions to reduce hospital admissions.

## Introduction

Multimorbidity is defined as the coexistence of two or more chronic diseases in an individual [1]. As the elderly population increases, the number of patients with multimorbidity is expected to rise [2]. In Japan, it has already been reported that over 60% of people aged 65 and older have multimorbidity [3]. Additionally, reports have linked multimorbidity to various adverse events [4]. The increase in the number of comorbid conditions leads to a rise in the involvement of multiple medical departments, an increased economic burden, and a heightened risk of physical function decline, progression of frailty, and decreased quality of life [5]. Furthermore, multimorbidity is associated with worse outcomes, including discharge to long-term care facilities, emergency room visits, higher hospitalization rates, and increased mortality [5]. Moreover, studies on the epidemiology of multiple diseases in the elderly suggest that baseline chronic disability has a more significant impact on physical function and survival than the number of comorbid diseases [6].

General medicine plays a pivotal role as a representative specialty in addressing the complexities of multimorbidity, and improving the quality of care in this context is essential [7,8]. In community hospitals, where elderly patients are prevalent, multimorbidity is common [9]. By appropriately assessing multimorbidity in primary care, it is possible to predict outcomes related to multimorbidity, such as hospitalization, physical function decline, and mortality [10]. Additionally, by accurately assessing the severity of multimorbidity, the degree of impact on the prognosis for each condition can be understood [5]. This, in turn, facilitates management that prioritizes disease treatment and is expected to reduce the provision of excessive medical care.

The concept of chronic organ disorder may be crucial in explaining multimorbidity. One method to represent multimorbidity is chronic organ disorder, which refers to the chronic conditions of major organs, heart, lungs, kidneys, etc., and is a concept proposed in Japan, increasingly applied to address chronic diseases in the elderly [11]. Specifically, chronic organ disorder encompasses the classical four major organ disorders (heart, lungs, liver, kidneys) and two additional disorders (neurological, musculoskeletal) that comprise a substantial portion of geriatric syndromes in a super-aged society [11]. This framework allows for an overall assessment of a patient's condition before delving into specific diseases, enabling the identification of problems, prioritization, and prognosis prediction in clinical practice. The evaluation of particular organ disorders is as follows: Stage A, where only risk factors are recognized; Stage B, where structural abnormalities in organs are observed; Stage C, where clinical symptoms or abnormal test results necessitate treatment; Stage D, where repeated acute exacerbations lead to decompensated function; and Stage E, where the patient is in the terminal stage with a prognosis of less than six months [11]. Utilizing this categorization to manage multimorbidity allows for tailored patient care.

No studies have clarified the relationship between chronic organ disorders and medical outcomes. One of the medical outcomes considered in this study is the hospitalization rate, commonly used in academic literature and regarded as an outcome related to multimorbidity [12]. This study aims to scientifically validate the use of chronic organ disorder by classifying elderly patients with multimorbidity based on chronic organ disorder and investigating its relationship with hospitalization rates. By identifying patients at higher risk of hospitalization due to their chronic organ disorder, the study seeks to strengthen preventive measures against hospitalization. Therefore, this research examines the relationship between chronic organ disorder and hospitalization in the general medicine outpatient clinic, where multimorbidity is most frequently managed.

## Materials and methods

This study retrospectively analyzed a cohort of patients who presented to the General Medicine Department at Unnan Municipal Hospital.

Setting

In 2022, Unnan City had a total population of 35,738, comprising 17,231 males and 18,507 females. Residents aged 65 years and older made up 40.27% of the population. The area was served by a single public hospital with 281 beds, categorized into 155 acute care, 48 general, 30 rehabilitation, and 48 chronic care beds. The Department of Family Medicine at this hospital coordinates the care of internal medicine patients in collaboration with various healthcare professionals [10].

Participants

The study population consisted of all patients who presented to the General Medicine Department at Unnan Municipal Hospital between April 2022 and September 2022. Inclusion criteria were patients aged 18 years and older, patients who had at least one visit to the General Medicine outpatient department during the study period, and patients with a documented diagnosis of two or more chronic diseases (multimorbidity), as defined by the presence of at least two chronic conditions, including but not limited to cardiovascular disease, chronic respiratory disease, diabetes, chronic kidney disease, or chronic neurological disorders. Exclusion criteria were patients who were younger than 18 years, patients with incomplete medical records or missing data relevant to the study, patients who only visited the clinic for a single acute condition without a documented chronic disease, and patients who were referred to other specialized departments without further follow-up in the General Medicine Department during the study period.

Measurements

Patient data including age, sex, chief complaint, medical history, diagnosis, treatment, hospitalization, eGFR, creatinine, hemoglobin, and serum albumin levels were measured.

Each organ disorder was classified into stages from A to E, with increasing severity from risk factors only (stage A) to end-stage with a prognosis of less than six months (stage E) [11]. This chronic organ disorder (COD) classification was adapted from our institution's original staging method for clinical use. While the original system includes five stages (A-E), stages C through E were consolidated to simplify the classification process (Table 1).

**Table 1:** Stage classification of chronic organ disorders. COPD: Chronic obstructive pulmonary diseases; AST: Aspartate aminotransferase; ALT: Alanine aminotransferase; γ-GTP: γ-glutamyl transpeptidase.

	Stage A (1)	Stage B (2)	Stage C and above (3)
Chronic heart disorder (CHD)	No history of heart diseases, Independent in daily living	A history of heart disease, Independent daily living	Active heart failure with dyspnea and/or edema
Chronic pulmonary disorder (CPD)	No history of lung diseases (COPD/interstitial pneumonia), independent in daily living	A history of lung diseases, independent in daily living	Active lung diseases with dyspnea
Chronic liver disorder (CLD)	No history of cirrhosis, No elevated liver enzymes (AST/ALT/γGTP)	No history of cirrhosis, Elevated liver enzymes on blood draw	Diagnosis of cirrhosis
Chronic renal disorder (CRD)	No history of chronic kidney disease, No renal function problems (eGFR≥60), No proteinuria on urinalysis	No history of chronic kidney disease, with renal dysfunction (eGFR<60) and/or proteinuria on urinalysis	History of chronic kidney diseases with renal dysfunction (eGFR<45)
Chronic brain disorder (CBD)	No diagnosis of dementia, Able to see a doctor alone	No diagnosis of dementia, With memory loss (medical questionnaire)	With the diagnosis of dementia
Chronic muscle disorder (CMD)	No knee osteoarthritis, No lumbar compression fracture, No osteoporosis, Able to be seen alone	Knee osteoarthritis, lumbar vertebra compression fracture, osteoporosis	Needs for family members with long-term care

Statistical analysis

For continuous variables, the normality of the data was tested before applying statistical tests. Parametric and nonparametric data were analyzed using Student's t-test and the Mann-Whitney U test, respectively. The chi-square test was used for categorical data. Multivariate logistic regression analysis was employed to examine the association between emergent admission and associated factors, including CODs. Multivariate logistic regression models were constructed using all variables known to be associated with the hospital admission rate and variables found to be significant in univariate regression models of hospital admission. All data analyses were performed using Easy R, version 1.23 (R Foundation for Statistical Computing, Vienna, Austria) [13]. Statistical significance was defined at P < 0.05.

Ethical considerations

The hospital ensured that patient anonymity and confidentiality of information would not be compromised. Information related to this study was posted on the hospital website without disclosing any details about the patients. In addition, contact information for hospital personnel was provided on the website to respond to questions regarding this study. All patients were informed about the purpose of this study, and informed consent was obtained. The Unnan City Hospital Clinical Ethics Committee approved the study protocol (#20230033).

## Results

The characteristics of the participants

The characteristics of the patients at baseline are summarized in Table 2. A total of 568 patients were included in the analysis, with 66 patients (11.6%) experiencing hospitalization during the study period. The hospitalized group’s mean age was significantly higher than that of the non-hospitalized group (75.52 ± 16.90 years vs. 58.77 ± 21.65 years, p < 0.001). The proportion of patients aged 75 and older was significantly higher among those hospitalized (62.1% vs. 26.1%, p < 0.001). Patients with higher stage COD were more likely to be hospitalized, with 47.0% of patients in the hospitalized group having COD stage B or C, compared to 21.6% in the non-hospitalized group (p < 0.001). Among the various organ disorders, CHD and CBD were significantly associated with hospitalization (p = 0.005 and p < 0.001, respectively). Compared to the non-hospitalized group, patients in the hospitalized group had lower serum albumin levels (3.70 ± 0.66 vs. 3.96 ± 0.39 g/dL, p < 0.001), lower BMI (20.65 ± 4.28 vs. 22.21 ± 3.92 kg/m², p = 0.003), higher serum creatinine levels (0.84 ± 0.47 vs. 0.71 ± 0.15 mg/dL, p < 0.001), a greater number of medications (5.92 ± 4.54 vs. 3.79 ± 4.30, p < 0.001), and a higher tendency to be dependent on others for medical visits (34.8% vs. 9.0%, p < 0.001) (Table 2).

**Table 2:** Baseline characteristics of the participants. BMI: Body mass index; eGFR: Estimated glomerular filtration rate; COD: Chronic organ disorder; CHD: Chronic heart disorder; CPD: Chronic pulmonary disorder; CLD: Chronic liver disorder; CRD: Chronic renal disorder; CBD: Chronic brain disorder; CMD: Chronic muscle disorder.

Factor	No Admission	Admission	Total	P-value
n	501	66	568	
Age, mean (SD)	58.77 (21.65)	75.52 (16.90)	60.75 (21.80)	<0.001
Age ≥75 (%)	131 (26.1)	41 (62.1)	173 (30.5)	<0.001
Male gender (%)	254 (50.7)	29 (43.9)	283 (49.8)	0.359
Hight, mean (SD)	160.39 (9.89)	157.64 (10.67)	160.05 (10.01)	0.035
Weight, mean (SD)	57.44 (12.87)	51.48 (12.39)	56.94 (12.63)	<0.001
BMI, mean (SD)	22.21 (3.92)	20.65 (4.28)	22.11 (3.85)	0.003
Albumin, mean (SD)	3.96 (0.39)	3.70 (0.66)	3.93 (0.44)	<0.001
Creatinine, mean (SD)	0.71 (0.15)	0.84 (0.47)	0.73 (0.22)	<0.001
eGFR, mean (SD)	68.15 (9.62)	63.40 (16.42)	67.61 (10.72)	0.001
Hemoglobin, mean (SD)	12.52 (1.31)	12.36 (2.47)	12.50 (1.49)	0.403
Medicine number, mean (SD)	3.79 (4.30)	5.92 (4.54)	4.08 (4.45)	<0.001
Medicine number ≥ 5 (%)	161 (33.8)	37 (56.1)	199 (35.0)	0.001
Dependent condition (%)	46 (9.2)	23 (34.8)	69 (12.2)	<0.001
COD (%)				
Stage A	393 (78.4)	35 (53.0)	428 (75.4)	<0.001
Stage B	77 (15.4)	16 (24.2)	94 (16.5)	
Stage C	31 (6.2)	15 (22.7)	46 (8.1)	
COD ≥ Stage B (%)	108 (21.6)	31 (47.0)	140 (24.6)	<0.001
CHD (%)				
Stage A	466 (93.2)	54 (81.8)	521 (91.9)	0.005
Stage B	25 (5.0)	8 (12.1)	33 (5.8)	
Stage C	9 (1.8)	4 (6.1)	13 (2.3)	
CPD (%)				
Stage A	487 (97.2)	61 (92.4)	549 (96.7)	0.071
Stage B	10 (2.0)	3 (4.5)	13 (2.3)	
Stage C	4 (0.8)	2 (3.0)	6 (1.1)	
CLD (%)				
Stage A	495 (98.8)	66 (100.0)	562 (98.9)	1
Stage B	4 (0.8)	0 (0.0)	4 (0.7)	
Stage C	2 (0.4)	0 (0.0)	2 (0.4)	
CRD (%)				
Stage A	490 (97.8)	66 (100.0)	557 (98.1)	0.728
Stage B	8 (1.6)	0 (0.0)	8 (1.4)	
Stage C	3 (0.6)	0 (0.0)	3 (0.5)	
CBD (%)				
Stage A	491 (98.0)	58 (87.9)	550 (96.8)	<0.001
Stage B	2 (0.4)	0 (0.0)	2 (0.4)	
Stage C	8 (1.6)	8 (12.1)	16 (2.8)	
CMD (%)				
Stage A	440 (87.8)	50 (75.8)	490 (86.3)	0.001
Stage B	54 (10.8)	10 (15.2)	65 (11.4)	
Stage C	7 (1.4)	6 (9.1)	13 (2.3)	

The logistic regression analysis for the factors associated with admission

Logistic regression analysis was performed to identify factors associated with chronic organ disorder in Stage B and above (Table 3). Based on the results from Table 2, variables considered to be associated with hospitalization were included. In this analysis, owing to the limited number of COD subcategories, we used COD as one independent variable to improve the study’s validity. Additionally, the logistic regression model selected variables that can be rapidly evaluated in an outpatient setting to demonstrate the utility of assessing patients’ clinical conditions during initial clinical visits. Multivariate logistic regression analysis identified age ≥75 years (OR 2.93, 95% CI 1.55-5.54, p < 0.001), COD stage ≥B (OR 1.86, 95% CI 1.01-3.45, p = 0.048), and lower BMI (OR 0.904, 95% CI 0.835-0.979, p = 0.013) as significant predictors of hospitalization (Table 3).

**Table 3:** Logistic regression analysis for the factors related to hospital admission. COD: Chronic organ disorder; CI: Confidence interval.

Factor	Odds ratio	Lower 95%CI	Upper 95%CI	P-value
Age ≥75	2.93	1.55	5.54	0.000918
Male gender	0.867	0.502	1.5	0.608
BMI	0.904	0.835	0.979	0.0129
Medicine number ≥ 5	1.25	0.661	2.36	0.494
COD ≥ Stage B	1.86	1.01	3.45	0.048

## Discussion

This study investigated the association between COD and hospitalization in patients with multimorbidity in a general medicine outpatient setting. The results demonstrate that the presence and severity of COD, particularly when involving two or more organ systems, significantly increase the risk of hospitalization. This aligns with previous research suggesting that organ disorder is a crucial predictor of adverse outcomes in multimorbid elderly populations.

One notable finding is the strong association between age and hospitalization rates. Patients aged 75 years and older were nearly three times more likely to be hospitalized compared to their younger counterparts. This is consistent with literature showing that advancing age correlates with both the number and severity of chronic conditions, which compound the likelihood of acute decompensations requiring hospitalization [6,14]. Moreover, this study adds to the body of evidence suggesting that frailty, functional decline, and other geriatric syndromes play a critical role in hospitalization risk, particularly when combined with COD. Older patients with COD can be at risk even with active activities of daily living (ADL) [15,16]. Thus, general physicians should take note of mild changes in their symptoms and approach them comprehensively [17].

The analysis also revealed significant associations between a comprehensive high COD stage, specific types of organ disorder, and hospitalization. Advanced COD (Stage B or above) was associated with a higher admission rate in the multivariate logistic regression model. Although the sample size restricted further analysis, CHD and CBD were particularly prominent in hospitalized patients, as shown by the univariate logistic regression model study. In particular, patients with advanced CHD (Stages C or above) exhibited higher hospitalization rates due to symptoms like dyspnea and edema, which are well-known precursors to acute exacerbations. Similarly, patients with advanced CBD (including those with dementia) demonstrated higher rates of hospitalization, potentially due to increased vulnerability to infections, falls, and medication mismanagement, as observed in other studies [18,19]. Heart failure and dementia are rising among older patients, and they may not disclose their symptoms [6,20]. General physicians should be vigilant for subtle changes in physical findings.

Another key finding is the inverse relationship between BMI and hospitalization, indicating that a lower BMI is associated with a higher risk of admission. This likely reflects the effects of undernutrition or muscle wasting, conditions commonly seen in patients with advanced multimorbidity, underscoring the importance of thorough nutritional assessments in this population [5,21,22]. The relationship between BMI and hospitalization is complex [23,24]. In obese patients, the prevalence of various diseases increases significantly. However, numerous studies have documented the 'obesity paradox,' where overweight and obese patients often have a better prognosis than those who are lean or underweight [25]. The results of the present study are consistent with these previous findings [25]. Among the older generation, as frailty is prevalent, this result can also reflect that frailty can manifest as low BMI and make older patients vulnerable to emergent admission to hospitals. General physicians should take care of patients with low BMI and frailty and approach them comprehensively to prevent further admissions.

The study has several clinical implications. First, the COD classification provides a valuable framework for assessing the risk of hospitalization in patients with multimorbidity. This categorization allows clinicians to identify high-risk patients and implement tailored interventions to prevent acute exacerbations. For example, more aggressive monitoring and early interventions for patients with Stage B or C disorders may reduce the likelihood of hospitalization through interprofessional collaboration in rural hospitals [26,27]. Furthermore, this approach can aid in prioritizing healthcare resources and targeting preventive strategies for those most in need [28].

Despite this study’s strengths, including its focus on a representative outpatient population and comprehensive assessment of multiple organ systems, there are some limitations. First, the retrospective design limits the ability to establish causality between COD and hospitalization. Additionally, the study population was drawn from a single center in Japan, and most participants were elderly patients, which may limit the generalizability of the findings to other settings or healthcare systems. Future studies could benefit from a prospective design and larger, more diverse populations to further validate these findings.

## Conclusions

This study demonstrates a strong association between COD stages and hospitalization rates among elderly patients with multimorbidity. Patients with advanced COD, particularly those with chronic heart and brain disorders, are at a significantly higher risk of hospitalization. Factors such as age, frailty, and low BMI further increase this risk. These findings underscore the importance of comprehensive multimorbidity assessments and early interventions to prevent hospitalizations. While the COD classification proves valuable in clinical practice, future research is necessary to validate these results across broader populations and healthcare settings.
